# 2,2′,4,4′,6,6′-Hexamethyl­biphenyl-3,3′,5,5′-tetra­yltetra­methyl­ene tetra­acetate

**DOI:** 10.1107/S1600536809018261

**Published:** 2009-05-20

**Authors:** Tuoping Hu

**Affiliations:** a Department of Chemistry, North University of China, Taiyuan, Shanxi 030051, People’s Republic of China

## Abstract

The title compound, C_30_H_38_O_8_, possess *C_i_* symmetry, with the inversion center situated at the center of the bridging C—C bond. In the crystal structure, mol­ecules are held together by C—H⋯O inter­actions.

## Related literature

For related structures, see: Frohlich & Musso (1985 ▶), Moorthy *et al.* (2002 ▶, 2005 ▶, 2006*a* ▶,*b* ▶); Natarajan *et al.* (2005*a* ▶,*b* ▶); Pickett (1936 ▶).
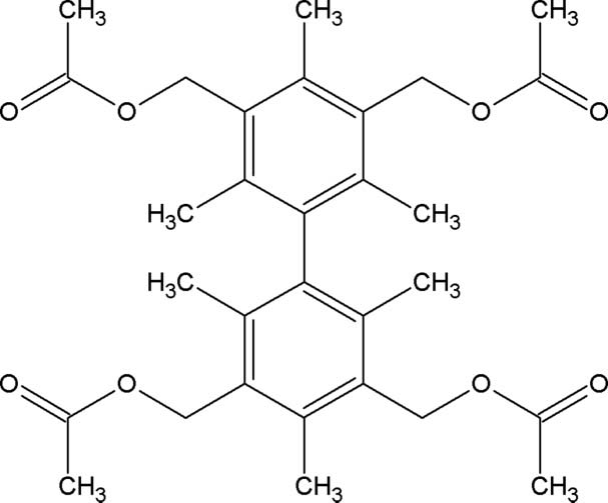


## Experimental

### 

#### Crystal data

C_30_H_38_O_8_
*M* *_r_* = 526.60
Orthorhombic, 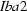

*a* = 15.336 (2) Å
*b* = 12.658 (1) Å
*c* = 14.755 (2) Å
*V* = 2864.3 (6) Å^3^
*Z* = 4
Mo *K*α radiation
μ = 0.09 mm^−1^
*T* = 293 K
0.20 × 0.10 × 0.10 mm

#### Data collection

Bruker SMART CCD area-detector diffractometer
Absorption correction: multi-scan (*SADABS*; Sheldrick, 1996 ▶) *T*_min_ = 0.983, *T*_max_ = 0.991
8052 measured reflections
1709 independent reflections
1045 reflections with *I* > 2σ(*I*)
*R*_int_ = 0.046

#### Refinement

*R*[*F* ^2^ > 2σ(*F* ^2^)] = 0.057
*wR*(*F* ^2^) = 0.199
*S* = 1.02
1709 reflections
173 parameters
2 restraints
H-atom parameters constrained
Δρ_max_ = 0.21 e Å^−3^
Δρ_min_ = −0.14 e Å^−3^

### 

Data collection: *SMART* (Bruker, 2007 ▶); cell refinement: *SAINT-Plus* (Bruker, 2007 ▶); data reduction: *SAINT-Plus*; program(s) used to solve structure: *SHELXS97* (Sheldrick, 2008 ▶); program(s) used to refine structure: *SHELXL97* (Sheldrick, 2008 ▶); molecular graphics: *SHELXTL* (Sheldrick, 2008 ▶); software used to prepare material for publication: *SHELXTL*.

## Supplementary Material

Crystal structure: contains datablocks global, I. DOI: 10.1107/S1600536809018261/su2107sup1.cif

Structure factors: contains datablocks I. DOI: 10.1107/S1600536809018261/su2107Isup2.hkl

Additional supplementary materials:  crystallographic information; 3D view; checkCIF report

## Figures and Tables

**Table 1:** Hydrogen-bond geometry (Å, °)

*D*—H⋯*A*	*D*—H	H⋯*A*	*D*⋯*A*	*D*—H⋯*A*
C15—H15*A*⋯O1^i^	0.96	2.58	3.472 (7)	155

Symmetry code: (i) 
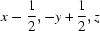
.

**Fig. 1.**
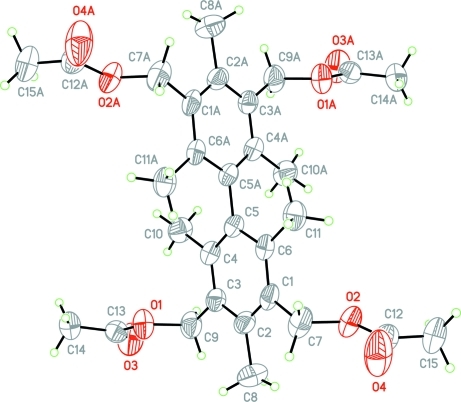
Molecular structure of the title compound, showing 50% probability displacement ellipsoids. The atoms marked with A are derived from the reference atoms by means of the symmetry transformation (1 -x, -y, z) .

**Fig. 2.**
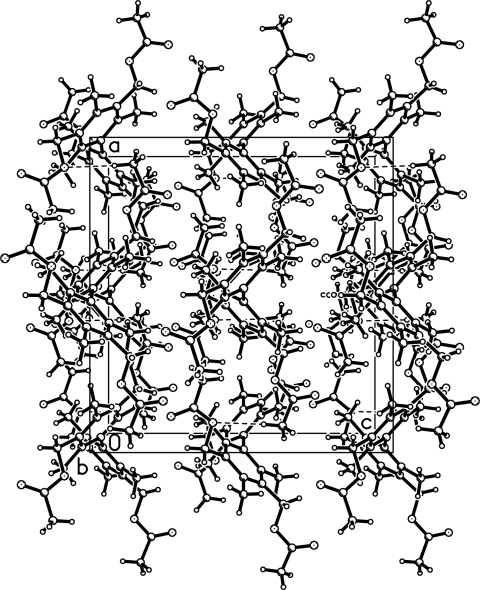
Crystal packing of the title compound viewed along the c axis.

## supplementary crystallographic information

### Comment

The title compound, illustrated in Fig. 1, was obtained as a byproduct when preparing the first order dendrimer by using 2,2',4,4',6,6'-hexamethyl-3,3',5,5''-biphenylene- tetramethanol. The molecule possesses a centre of inversion situated at the center of the bridging C-C bond. The two benzene rings are almost perpendicular to one another, with a dihedral angle of 82.71 (2) °. The geometry and bond distances are close to those observed in similar structures (Frohlich *et al.*, 1985); Moorthy *et al.*, 2002, 2005, 2006*a*,*b*; Natarajan *et al.*, 2005*a*,*b*; Pickett, 1936).

In the crystal structure of the title compound adjacent molecules have normal hydrophobic contacts with no intercalation or stacking interactions, only C-H···O interactions (Table 1 and Fig. 2).

### Experimental

To (2,2',4,4',6,6'-trimethyl-1,1',3,3'-phenylene) tetramethanol (10 mmol, 3.585 mg), in 50 ml of CH_3_COOH, was added 5 g of KOH. The mixture was stirred and heated at reflux for 24 h. The solution was then filtered, and the filtrate concentrated under vacumn. The sticky solid obtained was recrystalized in a mixture of benzene and acetone (1:1). Colourless prismatic crystals of the title compound were obtained. They were filtered, washed with cool diethylether and air dried.

### Refinement

In the final cycles of refinement, in the absence of significant anomalous scattering effects, the 1464 Friedel pairs were merged and Δf" set to zero. All of the H atoms were positioned geometrically [C—H = 0.960 - 0.970 Å] and refined using a riding model [*U*_iso_(H) = 1.2 or 1.5*U*_eq_(C)].

### Crystal data

**Table:**

C_30_H_38_O_8_	*F*(000) = 1128
*M**_r_* = 526.60	*D*_x_ = 1.221 Mg m^−^^3^
Orthorhombic, *I**b**a*2	Mo *K*α radiation, λ = 0.71073 Å
Hall symbol: I 2 -2 c	Cell parameters from 1604 reflections
*a* = 15.336 (2) Å	θ = 2.3–22.7°
*b* = 12.658 (1) Å	µ = 0.09 mm^−^^1^
*c* = 14.755 (2) Å	*T* = 293 K
*V* = 2864.3 (6) Å^3^	Prism, colorless
*Z* = 4	0.20 × 0.10 × 0.10 mm

### Data collection

**Table:**

Bruker SMART CCD area-detector diffractometer	1709 independent reflections
Radiation source: fine-focus sealed tube	1045 reflections with *I* > 2σ(*I*)
graphite	*R*_int_ = 0.046
φ and ω scans	θ_max_ = 27.6°, θ_min_ = 2.7°
Absorption correction: multi-scan (*SADABS*; Sheldrick, 1996)	*h* = −19→17
*T*_min_ = 0.983, *T*_max_ = 0.991	*k* = −16→12
8052 measured reflections	*l* = −19→18

### Refinement

**Table:**

Refinement on *F*^2^	Primary atom site location: structure-invariant direct methods
Least-squares matrix: full	Secondary atom site location: difference Fourier map
*R*[*F*^2^ > 2σ(*F*^2^)] = 0.057	Hydrogen site location: inferred from neighbouring sites
*wR*(*F*^2^) = 0.199	H-atom parameters constrained
*S* = 1.01	*w* = 1/[σ^2^(*F*_o_^2^) + (0.1286*P*)^2^] where *P* = (*F*_o_^2^ + 2*F*_c_^2^)/3
1709 reflections	(Δ/σ)_max_ < 0.001
173 parameters	Δρ_max_ = 0.21 e Å^−^^3^
2 restraints	Δρ_min_ = −0.14 e Å^−^^3^

### Special details

**Table:**

Geometry. Bond distances, angles etc. have been calculated using the rounded fractional coordinates. All su's are estimated from the variances of the (full) variance-covariance matrix. The cell esds are taken into account in the estimation of distances, angles and torsion angles
Refinement. Refinement of *F*^2^ against ALL reflections. The weighted *R*-factor *wR* and goodness of fit *S* are based on *F*^2^, conventional *R*-factors *R* are based on *F*, with *F* set to zero for negative *F*^2^. The threshold expression of *F*^2^ > σ(*F*^2^) is used only for calculating *R*-factors(gt) *etc*. and is not relevant to the choice of reflections for refinement. *R*-factors based on *F*^2^ are statistically about twice as large as those based on *F*, and *R*- factors based on ALL data will be even larger.

### Fractional atomic coordinates and isotropic or equivalent isotropic displacement parameters (Å^2^)

**Table:**

	*x*	*y*	*z*	*U*_iso_*/*U*_eq_	
O1	0.5866 (2)	0.3433 (3)	−0.0969 (2)	0.0833 (12)	
O2	0.22394 (19)	0.1664 (3)	0.1133 (2)	0.0781 (12)	
O3	0.6098 (3)	0.4057 (4)	−0.2354 (3)	0.1157 (19)	
O4	0.1730 (3)	0.2011 (6)	0.2488 (4)	0.140 (2)	
C1	0.3745 (2)	0.1758 (4)	0.0700 (3)	0.0633 (13)	
C2	0.4014 (3)	0.2522 (4)	0.0068 (4)	0.0670 (16)	
C3	0.4673 (3)	0.2301 (3)	−0.0546 (3)	0.0627 (14)	
C4	0.5069 (3)	0.1309 (3)	−0.0540 (3)	0.0595 (11)	
C5	0.4793 (2)	0.0532 (3)	0.0067 (3)	0.0542 (11)	
C6	0.4117 (2)	0.0748 (4)	0.0680 (2)	0.0565 (13)	
C7	0.3093 (3)	0.2018 (5)	0.1427 (4)	0.0867 (18)	
C8	0.3548 (5)	0.3593 (5)	0.0064 (7)	0.116 (3)	
C9	0.4985 (4)	0.3108 (4)	−0.1239 (4)	0.0833 (16)	
C10	0.5800 (4)	0.1052 (4)	−0.1200 (4)	0.0813 (17)	
C11	0.3798 (3)	−0.0116 (5)	0.1307 (3)	0.0773 (16)	
C12	0.1610 (3)	0.1719 (4)	0.1736 (3)	0.0737 (17)	
C13	0.6365 (3)	0.3889 (4)	−0.1602 (4)	0.0687 (17)	
C14	0.7248 (3)	0.4091 (5)	−0.1300 (4)	0.088 (2)	
C15	0.0765 (3)	0.1341 (6)	0.1374 (4)	0.096 (2)	
H7A	0.32520	0.16690	0.19880	0.1040*	
H7B	0.30840	0.27740	0.15330	0.1040*	
H8A	0.35990	0.39140	0.06510	0.1730*	
H8B	0.29430	0.34920	−0.00790	0.1730*	
H8C	0.38110	0.40440	−0.03820	0.1730*	
H9A	0.49950	0.27960	−0.18400	0.1000*	
H9B	0.45970	0.37130	−0.12480	0.1000*	
H10A	0.59200	0.16590	−0.15690	0.1220*	
H10B	0.56270	0.04730	−0.15800	0.1220*	
H10C	0.63150	0.08610	−0.08670	0.1220*	
H11A	0.41300	−0.07480	0.12030	0.1160*	
H11B	0.31930	−0.02530	0.11910	0.1160*	
H11C	0.38710	0.01040	0.19250	0.1160*	
H14A	0.73100	0.38720	−0.06810	0.1320*	
H14B	0.73710	0.48320	−0.13490	0.1320*	
H14C	0.76490	0.37020	−0.16720	0.1320*	
H15A	0.08300	0.11650	0.07440	0.1430*	
H15B	0.03350	0.18860	0.14380	0.1430*	
H15C	0.05830	0.07250	0.17040	0.1430*	

### Atomic displacement parameters (Å^2^)

**Table:**

	*U*^11^	*U*^22^	*U*^33^	*U*^12^	*U*^13^	*U*^23^
O1	0.067 (2)	0.109 (2)	0.074 (2)	−0.0219 (18)	−0.0067 (17)	0.0083 (19)
O2	0.0487 (14)	0.123 (3)	0.0627 (17)	0.0076 (16)	0.0032 (14)	−0.0154 (17)
O3	0.100 (3)	0.149 (4)	0.098 (3)	−0.014 (3)	−0.013 (3)	0.043 (3)
O4	0.098 (3)	0.226 (6)	0.096 (3)	−0.051 (3)	0.036 (3)	−0.067 (4)
C1	0.0368 (18)	0.090 (3)	0.063 (2)	0.0000 (19)	−0.0031 (17)	−0.021 (2)
C2	0.054 (2)	0.069 (3)	0.078 (3)	0.007 (2)	−0.009 (2)	−0.010 (2)
C3	0.052 (2)	0.074 (3)	0.062 (2)	−0.0011 (19)	−0.008 (2)	0.002 (2)
C4	0.0474 (19)	0.079 (2)	0.052 (2)	0.0014 (19)	0.0024 (17)	−0.0007 (19)
C5	0.0443 (19)	0.068 (2)	0.0502 (19)	0.0035 (16)	−0.0030 (16)	0.0004 (19)
C6	0.0395 (17)	0.082 (3)	0.048 (2)	−0.0061 (18)	0.0007 (15)	−0.0087 (19)
C7	0.057 (2)	0.124 (4)	0.079 (3)	0.003 (3)	0.001 (2)	−0.033 (3)
C8	0.097 (4)	0.085 (4)	0.165 (7)	0.033 (3)	−0.005 (5)	−0.007 (4)
C9	0.066 (2)	0.093 (3)	0.091 (3)	−0.010 (3)	−0.015 (2)	0.023 (3)
C10	0.076 (3)	0.092 (3)	0.076 (3)	0.006 (2)	0.026 (3)	0.007 (3)
C11	0.063 (2)	0.100 (3)	0.069 (3)	−0.007 (3)	0.015 (2)	0.003 (3)
C12	0.069 (3)	0.091 (3)	0.061 (3)	0.002 (2)	0.011 (2)	−0.007 (2)
C13	0.076 (3)	0.067 (3)	0.063 (3)	0.001 (2)	0.000 (2)	0.005 (2)
C14	0.072 (3)	0.102 (4)	0.090 (4)	−0.017 (3)	−0.002 (3)	−0.003 (3)
C15	0.060 (3)	0.151 (5)	0.076 (3)	−0.005 (3)	0.007 (2)	0.002 (3)

### Geometric parameters (Å, °)

**Table:**

O1—C9	1.468 (7)	C7—H7A	0.9700
O1—C13	1.338 (6)	C7—H7B	0.9700
O2—C7	1.450 (6)	C8—H8A	0.9600
O2—C12	1.315 (5)	C8—H8B	0.9600
O3—C13	1.202 (7)	C8—H8C	0.9600
O4—C12	1.184 (8)	C9—H9A	0.9700
C1—C2	1.405 (7)	C9—H9B	0.9700
C1—C6	1.400 (7)	C10—H10A	0.9600
C1—C7	1.503 (7)	C10—H10B	0.9600
C2—C3	1.386 (7)	C10—H10C	0.9600
C2—C8	1.533 (8)	C11—H11A	0.9600
C3—C4	1.395 (6)	C11—H11B	0.9600
C3—C9	1.523 (7)	C11—H11C	0.9600
C4—C5	1.396 (6)	C14—H14A	0.9600
C4—C10	1.520 (7)	C14—H14B	0.9600
C5—C6	1.403 (5)	C14—H14C	0.9600
C5—C5^i^	1.489 (5)	C15—H15A	0.9600
C6—C11	1.514 (7)	C15—H15B	0.9600
C12—C15	1.481 (7)	C15—H15C	0.9600
C13—C14	1.448 (7)		
			
O1···C10	3.035 (6)	H7A···C11	2.6100
O2···C11	3.295 (6)	H7A···H11C	2.2000
O3···C7^ii^	3.382 (8)	H7A···H10A^vii^	2.4800
O4···C9^iii^	3.236 (8)	H7B···O4	2.6900
O1···H15A^iv^	2.5800	H7B···C8	2.5100
O1···H10A	2.4200	H7B···H8A	2.1000
O2···H11B	2.8300	H7B···H8B	2.5600
O2···H14A^v^	2.7600	H7B···O3^vii^	2.6300
O3···H9A	2.4500	H8A···C7	2.7700
O3···H7B^ii^	2.6300	H8A···H7B	2.1000
O3···H15C^vi^	2.6500	H8B···C7	2.9100
O3···H9B	2.8600	H8B···H7B	2.5600
O4···H7B	2.6900	H8C···C9	2.5000
O4···H14C^vii^	2.6500	H8C···H9B	1.8100
O4···H7A	2.4900	H9A···O3	2.4500
O4···H9A^iii^	2.8400	H9A···C10	2.7000
O4···H9B^iii^	2.9100	H9A···H10A	2.0600
C4···C10^i^	3.414 (7)	H9A···O4^viii^	2.8400
C4···C11^i^	3.567 (7)	H9A···C15^viii^	3.0800
C6···C11^i^	3.424 (6)	H9B···O3	2.8600
C6···C10^i^	3.592 (7)	H9B···C8	2.5200
C7···O3^vii^	3.382 (8)	H9B···H8C	1.8100
C9···O4^viii^	3.236 (8)	H9B···O4^viii^	2.9100
C10···C4^i^	3.414 (7)	H10A···O1	2.4200
C10···C6^i^	3.592 (7)	H10A···C9	2.3800
C10···O1	3.035 (6)	H10A···C13	2.9000
C11···C4^i^	3.567 (7)	H10A···H9A	2.0600
C11···O2	3.295 (6)	H10A···H7A^ii^	2.4800
C11···C6^i^	3.424 (6)	H10B···C4^i^	2.9300
C2···H15B^iv^	2.9600	H10B···C5^i^	2.8200
C4···H11A^i^	2.9400	H10B···C10^i^	2.9700
C4···H10B^i^	2.9300	H10B···H10B^i^	2.2700
C5···H11A^i^	2.3700	H10B···H11C^ii^	2.3800
C5···H10B^i^	2.8200	H10C···C5^i^	2.8100
C5···H10C^i^	2.8100	H10C···H14B^ix^	2.5000
C6···H11A^i^	2.8000	H11A···C4^i^	2.9400
C7···H11C	2.8000	H11A···C5^i^	2.3700
C7···H8A	2.7700	H11A···C6^i^	2.8000
C7···H8B	2.9100	H11B···O2	2.8300
C7···H11B	2.9000	H11B···C7	2.9000
C8···H9B	2.5200	H11C···C7	2.8000
C8···H7B	2.5100	H11C···H7A	2.2000
C9···H10A	2.3800	H11C···C10^vii^	3.0600
C9···H8C	2.5000	H11C···H10B^vii^	2.3800
C10···H10B^i^	2.9700	H14A···O2^iv^	2.7600
C10···H11C^ii^	3.0600	H14B···H10C^x^	2.5000
C10···H9A	2.7000	H14C···O4^ii^	2.6500
C11···H7A	2.6100	H15A···O1^v^	2.5800
C13···H10A	2.9000	H15B···C2^v^	2.9600
C15···H9A^iii^	3.0800	H15C···H15C^xi^	2.5600
H7A···O4	2.4900	H15C···O3^xii^	2.6500
			
C9—O1—C13	117.3 (4)	C2—C8—H8B	109.00
C7—O2—C12	116.4 (4)	C2—C8—H8C	109.00
C2—C1—C6	119.7 (4)	H8A—C8—H8B	109.00
C2—C1—C7	121.2 (5)	H8A—C8—H8C	109.00
C6—C1—C7	119.1 (4)	H8B—C8—H8C	110.00
C1—C2—C3	120.6 (4)	O1—C9—H9A	110.00
C1—C2—C8	118.3 (5)	O1—C9—H9B	110.00
C3—C2—C8	121.1 (5)	C3—C9—H9A	110.00
C2—C3—C4	119.7 (4)	C3—C9—H9B	110.00
C2—C3—C9	122.2 (4)	H9A—C9—H9B	109.00
C4—C3—C9	118.1 (4)	C4—C10—H10A	109.00
C3—C4—C5	120.4 (4)	C4—C10—H10B	109.00
C3—C4—C10	120.6 (4)	C4—C10—H10C	109.00
C5—C4—C10	118.9 (4)	H10A—C10—H10B	109.00
C4—C5—C6	120.0 (4)	H10A—C10—H10C	110.00
C4—C5—C5^i^	120.5 (3)	H10B—C10—H10C	109.00
C5^i^—C5—C6	119.4 (4)	C6—C11—H11A	109.00
C1—C6—C5	119.5 (4)	C6—C11—H11B	110.00
C1—C6—C11	121.0 (3)	C6—C11—H11C	109.00
C5—C6—C11	119.5 (4)	H11A—C11—H11B	109.00
O2—C7—C1	108.6 (4)	H11A—C11—H11C	109.00
O1—C9—C3	107.2 (4)	H11B—C11—H11C	110.00
O2—C12—O4	122.5 (5)	C13—C14—H14A	110.00
O2—C12—C15	112.4 (4)	C13—C14—H14B	109.00
O4—C12—C15	125.1 (5)	C13—C14—H14C	109.00
O1—C13—O3	121.7 (5)	H14A—C14—H14B	110.00
O1—C13—C14	113.3 (5)	H14A—C14—H14C	109.00
O3—C13—C14	124.9 (5)	H14B—C14—H14C	109.00
O2—C7—H7A	110.00	C12—C15—H15A	109.00
O2—C7—H7B	110.00	C12—C15—H15B	109.00
C1—C7—H7A	110.00	C12—C15—H15C	109.00
C1—C7—H7B	110.00	H15A—C15—H15B	109.00
H7A—C7—H7B	108.00	H15A—C15—H15C	109.00
C2—C8—H8A	109.00	H15B—C15—H15C	110.00
			
C13—O1—C9—C3	160.9 (4)	C8—C2—C3—C9	1.5 (8)
C9—O1—C13—O3	2.2 (7)	C2—C3—C4—C5	1.4 (7)
C9—O1—C13—C14	−174.5 (4)	C2—C3—C4—C10	−179.3 (5)
C12—O2—C7—C1	172.3 (4)	C9—C3—C4—C5	−178.5 (4)
C7—O2—C12—O4	−2.1 (8)	C9—C3—C4—C10	0.9 (7)
C7—O2—C12—C15	−180.0 (5)	C2—C3—C9—O1	110.3 (5)
C6—C1—C2—C3	−3.1 (7)	C4—C3—C9—O1	−69.9 (5)
C6—C1—C2—C8	175.7 (5)	C3—C4—C5—C6	−0.4 (6)
C7—C1—C2—C3	174.1 (4)	C3—C4—C5—C5^i^	179.2 (4)
C7—C1—C2—C8	−7.1 (7)	C10—C4—C5—C6	−179.7 (4)
C2—C1—C6—C5	4.1 (6)	C10—C4—C5—C5^i^	−0.1 (6)
C2—C1—C6—C11	−175.7 (4)	C4—C5—C6—C1	−2.3 (5)
C7—C1—C6—C5	−173.2 (4)	C4—C5—C6—C11	177.4 (4)
C7—C1—C6—C11	7.1 (6)	C5^i^—C5—C6—C1	178.1 (3)
C2—C1—C7—O2	98.1 (5)	C5^i^—C5—C6—C11	−2.2 (5)
C6—C1—C7—O2	−84.7 (5)	C4—C5—C5^i^—C4^i^	−83.7 (5)
C1—C2—C3—C4	0.4 (7)	C4—C5—C5^i^—C6^i^	95.9 (5)
C1—C2—C3—C9	−179.8 (5)	C6—C5—C5^i^—C4^i^	95.9 (5)
C8—C2—C3—C4	−178.4 (5)	C6—C5—C5^i^—C6^i^	−84.5 (4)

Symmetry codes: (i) −*x*+1, −*y*, *z*; (ii) −*x*+1, *y*, *z*−1/2; (iii) −*x*+1/2, −*y*+1/2, *z*+1/2; (iv) *x*+1/2, −*y*+1/2, *z*; (v) *x*−1/2, −*y*+1/2, *z*; (vi) *x*+1/2, *y*+1/2, *z*−1/2; (vii) −*x*+1, *y*, *z*+1/2; (viii) −*x*+1/2, −*y*+1/2, *z*−1/2; (ix) −*x*+3/2, *y*−1/2, *z*; (x) −*x*+3/2, *y*+1/2, *z*; (xi) −*x*, −*y*, *z*; (xii) *x*−1/2, *y*−1/2, *z*+1/2.

### Hydrogen-bond geometry (Å, °)

**Table:**

*D*—H···*A*	*D*—H	H···*A*	*D*···*A*	*D*—H···*A*
C10—H10A···O1	0.96	2.42	3.035 (6)	122
C15—H15A···O1^v^	0.96	2.58	3.472 (7)	155

Symmetry codes: (v) *x*−1/2, −*y*+1/2, *z*.
